# Stair-Walking Performance in Adolescents with Intellectual Disabilities

**DOI:** 10.3390/s16071066

**Published:** 2016-07-11

**Authors:** Wann-Yun Shieh, Yan-Ying Ju, Yu-Chun Yu, Che-Kuan Lin, Yen-Tzu Lin, Hsin-Yi Kathy Cheng

**Affiliations:** 1 Department of Computer Science and Information Engineering, Chang Gung University, No. 259, Wen-Hwa 1st Road, Kwei-Shan, Tao-Yuan 333, Taiwan; wyshieh@mail.cgu.edu.tw; 2 Department of Adapted Physical Education, National Taiwan Sport University, No. 250, Wen-Hwa 1st Road, Kwei-Shan, Tao-Yuan 333, Taiwan; yanju@ntsu.edu.tw; 3 Department of Rehabilitation, National Taoyuan Special School, Tao-Yuan 330, Taiwan; ot.spring.yu@gmail.com; 4 Graduate Institute of Medical Mechatronics, Chang Gung University, No. 259, Wen-Hwa 1st Road, Kwei-Shan, Tao-Yuan 333, Taiwan; aa1030520023@gmail.com; 5 Graduate Institute of Early Intervention, Chang Gung University, No. 259, Wen-Hwa 1st Road, Kwei-Shan, Tao-Yuan 333, Taiwan; vickiy7711@gmail.com; 6 Department of Physical Medicine and Rehabilitation, Chang Gung Memorial Hospital, 5 Fu-Hsing Street, Kwei-Shan, Tao-Yuan 333, Taiwan

**Keywords:** intellectual disabilities, mobility, gait, stair-walking, motion sensor

## Abstract

Most individuals with intellectual disabilities (ID) demonstrate problems in learning and movement coordination. Consequently, they usually have difficulties in activities such as standing, walking, and stair climbing. To monitor the physical impairments of these children, regular gross motor evaluation is crucial. Straight-line level walking is the most frequently used test of their mobility. However, numerous studies have found that unless the children have multiple disabilities, no significant differences can be found between the children with ID and typically-developed children in this test. Stair climbing presents more challenges than level walking because it is associated with numerous physical factors, including lower extremity strength, cardiopulmonary endurance, vision, balance, and fear of falling. Limited ability in those factors is one of the most vital markers for children with ID. In this paper, we propose a sensor-based approach for measuring stair-walking performance, both upstairs and downstairs, for adolescents with ID. Particularly, we address the problem of sensor calibration to ensure measurement accuracy. In total, 62 participants aged 15 to 21 years, namely 32 typically-developed (TD) adolescents, 20 adolescents with ID, and 10 adolescents with multiple disabilities (MD), participated. The experimental results showed that stair-walking is more sensitive than straight-line level walking in capturing gait characteristics for adolescents with ID.

## 1. Introduction

Statistics revealed that 2% of the world’s population lives with intellectual disabilities (ID) [1]. Most individuals with ID demonstrate problems in learning and motor control [2]. Consequently, they usually have difficulties in activities that require movement coordination, such as standing, walking, and stair climbing [3,4,5,6].

To monitor the physical performance of these children, regular gross motor evaluation is vital. Among the frequently used tests, tests of walking ability, especially straight-line level walking, are the most pivotal for measuring mobility [3,7,8,9,10,11,12]. However, Iosa et al. (2014) [7] showed that unless the adolescents have multiple disabilities, such as cognitive or physical problems, no significant differences can be found between adolescents with ID and typically developed (TD) adolescents in a straight-line level walking test. This is reasonable because, compared with other complicated tasks, such as obstacle crossing, straight-line level walking is less challenging and generally requires less lower extremity muscle control and strength [13,14]. The obstacle crossing test is a standardized criterion-based evaluation that is often used in numerous motor performance tests. However, this test is not adequate when used for participants with ID because it typically requires the subject’s attention and active participation, which most participants with ID lack. Moreover, if the motion being evaluated is not a familiar task, most of the participants with ID experience difficulty in following the testing instructions. Consequently, tracking the motor performance of these participants presents a great challenge.

**Figure 1:**
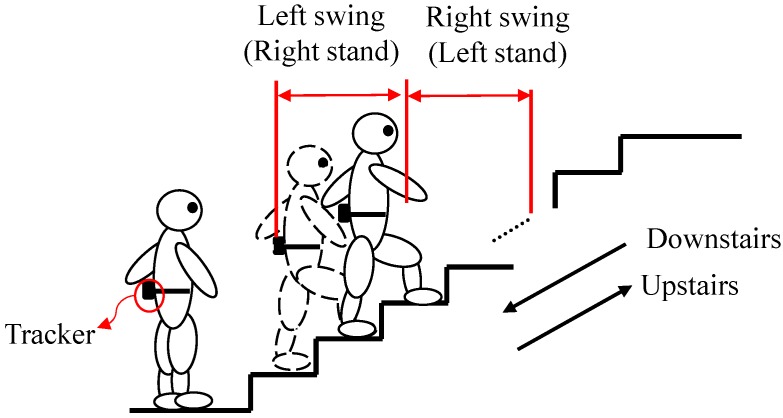
Upstairs and downstairs stair-walking.

Another mobility test, stair-walking, is also a crucial functional motor evaluation for children and adolescents with ID [14,15,16,17]. Like normal gait walking, stair-walking comprises stance phases (weight acceptance, forward continuance, and controlled lowering) and swing phases (leg pull-through and preparation of foot placement) alternatively performed by the lower limbs [15,16], as shown in Figure 1. To ascend or descend stairs safely, a person must resist the force of gravity by contracting the rectus femoris, vastus lateralis, soleus, and medial gastrocnemius during two phases [16]. Stair-walking is an everyday skill for children and adolescents with ID and the objective is clearly understood [17]. Such a test presents numerous challenges to participants with ID [8,14,15] because stair-walking is associated with factors including lower extremity strength, cardiopulmonary endurance, vision, balance, and fear of falling. Limited ability in those factors is one of the most crucial markers for participants with ID [15]. To prevent postural instability or falls during stair-walking, children with ID often develop unusual motion patterns, such as tap-stepping and forward leaning [16].

Currently, studies on stair-walking tests for children and adolescents with ID are limited. Most studies can be partitioned into two approaches. The first approach is to evaluate stair ability through expert scoring, an example of which is the Dynamic Gait Index (DGI) [18]. The final testing item of the DGI concerns stair-climbing performance, and the score is determined according to a therapist’s observation, ranging from 0–3 points. However, the score represents only an overall description of stair-climbing performance (e.g., walking with or without a rail). Pattern changes and movement details cannot be measured objectively through expert scoring. Therefore, numerous other studies have adopted the second approach, that is, using a motion capture system (i.e., based on optical cameras) and force plates to measure the gait; this has been considered the gold standard in the industry and at most hospitals [3,4,5,6,7,8,9]. Such systems can detect comprehensive temporal and spatial gait features. However, this approach has time and space limitations in stair-walking testing. For most participants with ID, even though a motion capture system can measure gait completely, the less limiting approach is to develop an easy-to-use, wearable, and portable tool that can be applied in daily or unrestricted-distance measurement for eliciting awareness of gait abnormalities throughout childhood and adulthood [10].

With the rapid development of the inertial sensor technology, numerous researchers have applied sensors based on microelectromechanical systems (MEMS) to measure gait motions [11,12,15,19,20,21,22]. The most widely used inertial sensors are accelerometers and gyroscopes. Accelerometers can be used to measure the acceleration of an object along its movement trajectory [11], and gyroscopes can be used to measure an object’s rotational angular velocity around three-dimensional axes (i.e., roll, pitch, and yaw) [12]. Both of them can be mounted on the limbs or the trunk to measure motions without undue restrictions of space or time. However, inertial sensors encounter accumulated drift effects [23,24] and gravity interventions [10]. These problems render the measurements inaccurate, which inherently limits the sensors’ applications. Therefore, most studies that use inertial sensors focus on measuring the temporal gait parameters, and use those parameters to explore various abnormalities prior to formal assessment, or investigate physical activity levels in children and youth with ID [25].

In this paper, we aim to propose an approach based on 3D accelerometers and gyroscopes for adolescents with ID to measure their stair-walking performance. In particular, we address the problem of sensor calibration to ensure accurate measurement. Rather than using only a straight-line level walking test, this study focused more on measuring the motor performance of children with ID through both upstairs and downstairs walking tests. We mounted an integrated sensor containing an accelerometer and a gyroscope on each participant’s lower back to measure their gait parameters. The testing protocol was approved by the Ethics Committee of Chang Gung Medical Foundation (Number: 103-7374B).

The remainder of this paper is organized as follows. In Section 2, we explain the proposed method and the testing procedure. In Section 3, we report and discuss the results. Finally, we present our conclusion in Section 4.

## 2. Materials and Methods

### 2.1. Participants

The experiment was performed in a special school in Northern Taiwan. A total of 62 participants aged 15–21 years participated in the experiment. On the basis of their clinical characteristics, they were divided into three groups: 32 TD adolescents (aged 17.97 ± 1.60 years), 20 adolescents with ID (aged 17.75 ± 0.97 years), and 10 adolescents with multiple disabilities (MD; aged 17.30 ± 0.67 years); their age ranges did not show any significant difference (*F* = 1.011, *p* = 0.370). Each child with MD had two or more areas of significant impairment; for example, ID with orthopedic, autistic, and/or other neurodevelopmental disorders. In this study, we screened the participants according to their activities of daily living (ADL), including transitioning from sitting to standing, transferring weight, obstacle crossing, selecting proper attire, dressing, and buttoning. A five-point score for each item was used, with a score of 5 indicating independent functional performance. Participants with ID had a total score of 28.25 ± 1.83, whereas those with MD had a total score of 26.20 ± 3.04. A significant difference was found between the two groups in ADL (*F* = 1.338, *p* = 0.029). Therefore, the adolescents with MD demonstrated reduced functional adaptive ability compared to those with ID. The number of adolescents with disabilities (ID and MD) was close to the number of TD adolescents. These adolescents with ID and MD were able to follow simple commands and walk without support, and had no serious heart or lung conditions. The test was supervised by a physical therapist and an assistant to ensure the safety of the children. Informed consent forms were signed by the participants and their legal guardians.

### 2.2. Procedure

**Figure 2:**
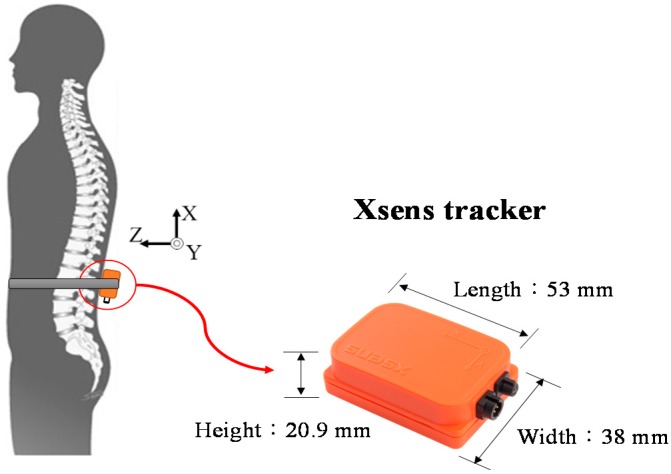
Xsens tracker and its position.

In this study, we used the Xsens tracker (Xsens, Enschede, Overijssel, The Netherlands) [26] for motion detection. Each Xsens tracker is an integrated sensor, composed of a three-axis accelerometer, a three-axis gyroscope, and a three-axis magnetometer. Only the data from the accelerometer and the gyroscope were used to detect stair-walking characteristics. The size of an Xsens tracker and the position at which it was mounted on a typical participant’s trunk are shown in Figure 2.

In each test, each participant was required to wear an elastic belt with an Xsens tracker on the lower back. The participants performed two tests: one was a 10-m straight-line level walking test, and the other was a stair-walking test comprising upstairs and downstairs components. A staircase with 13 steps (height and depth were both 15 cm) was used for the stair-walking test. Each participant started by walking downstairs. After 13 steps, the participant turned around and then walked upstairs for 13 steps. This was considered one round. Each participant repeated four rounds. The heart rate and the blood oxygen were monitored before and after each round to prevent any adverse events. A three minute rest was provided between each round to prevent fatigue. For data stability, the beginning and the end of the staircase were clipped, leaving the middle 10 steps for statistical analysis.

### 2.3. Signal Analysis

#### 2.3.1. Ten-Meter Straight-Line Level Walking

**Figure 3:**
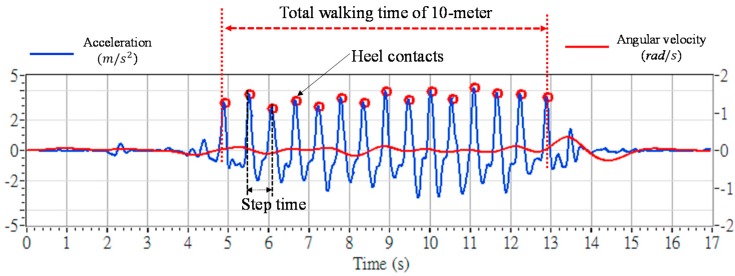
Signals from the accelerometer and the gyroscope in the 10-m walking test. The horizontal line represents time (s), the left vertical line shows the acceleration (m/s^2^) and the right vertical line shows the angular velocity (rad/s), respectively along and about the *X*-axis of the tracker.

Each participant performed the 10-m walking test first. Figure 3 shows signals collected by the accelerometer and the gyroscope from a TD adolescent, respectively along and about the X-axis of the tracker (i.e., corresponding to the up-and-down, and yaw direction in Figure 2). All signals were aligned on the basis of the same onset time. As shown in Figure 3, the accelerometer signals exhibit very clear pulses, and each pulse represents the pattern of one walking step. Meanwhile, the waveform of the angular velocity shows less variety because most participants (TD and ID) were able to keep their trunks stable and vertical without twists during level walking.

Since we mounted the sensor on the lower back of the trunk, each peak point in Figure 3 can be identified as the time that a heel (either left or right) contacted the ground [27]. From this observation, we can identify the gait events and use the peak points to derive two parameters:

(1) Step time: the time period between two sequential peak points in the waveform.
(2) Total walking time: the time required to finish the whole 10-m walking test, which can be calculated by summing all step times.

#### 2.3.2. Stair-Walking

**Figure 4:**
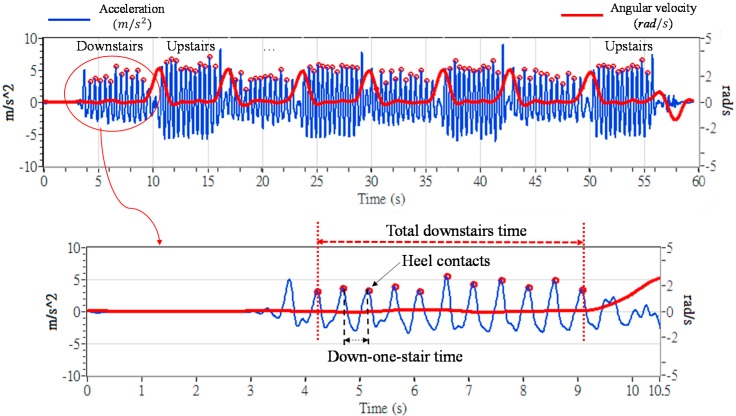
Upstairs and downstairs signals. The horizontal line represents time (s), the left vertical line shows the acceleration (m/s^2^), and the right vertical line shows the angular velocity (rad/s), respectively along and about the *X*-axis of the tracker.

Figure 4 shows the signals obtained from a TD participant. We can identify several features from the figure. First, we can use the gyroscope signals to divide the acceleration signals into upstairs and downstairs phases. The positive or negative peaks of the gyroscope represent the turning of the child at each stair platform. Second, the acceleration signals exhibit features such as ambulation amplitudes and frequencies during upstairs and downstairs phases. Third, the peak point of each acceleration pulse represents the time that a foot contacted a step. The segment between any two sequential peak points thus represents the time a child required to walk up or down a step (i.e., “up-one-step time” and “down-one-step time”). The total duration of upstairs and downstairs motion can be measured by summarizing the time in the upstairs and downstairs phases (i.e., “total upstairs time” and “total downstairs time”).

#### 2.3.3. Signal Processing

In this study, we used LabVIEW (National Instruments Corporation, Austin, TX, USA) to calculate and analyze all data. The process was divided into two phases. The first phase was the preprocessing of the signals, including the elimination of the influences of the gravity problem and the drift problem. The second phase was the detection of the features in the waveform. The details of each phase are shown as follows.

**Figure 5:**
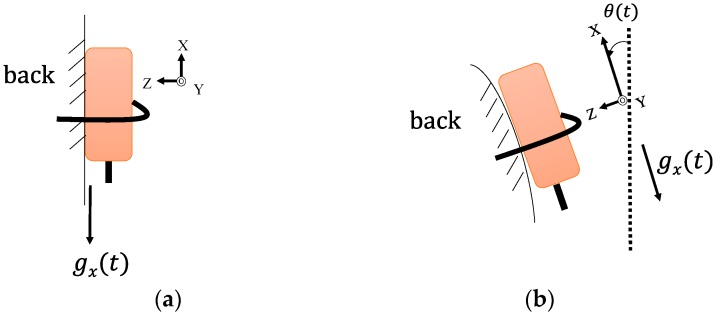
Adjusting for the influence of gravity. The *X*-axis is a local axis of the tracker, and $g_{x}\left(t\right)$ is defined along the *X*-axis. (**a**) the tracker is worn vertically; (**b**) the tracker is worn with an inclination angle θ(t) in plane X-Z.

The output of the accelerometer is composed of the “free” acceleration of the sensor itself and the intervention of gravity on the sensor. We take Figure 5 for an example. Figure 5a shows that if the tracker is worn vertically, gravity will affect the *X*-axis by 1 g (i.e., $9.8\text{ m}/s^{2}$). If the tracker is worn with an inclination angle θ(t) in plane X-Z, as shown in Figure 5b, gravity will have a component on the *X*-axis by $g_{x}\left(t\right)$. Then we obtain:

(1) $${a_{x}}^{\prime }\left(t\right)=a_{x}\left(t\right)+g_{x}\left(t\right)=a_{x}\left(t\right)+cos\theta \left(t\right)*G$$

where $a_{x}\left(t\right)$ is the free acceleration of the sensor along the *X*-axis (cf. Figure 5), ${a_{x}}_{\prime }\left(t\right)$ is the acceleration output of the tracker along the *X*-axis, and *G* is the module of the gravity vector *g*, which has been defined in [28]. Theoretically, the inclinations of the tracker on the plane X-Z and plane X-Y will affect $g_{x}\left(t\right).$ In practice, when the subject walked along a straight line or performed stair-walking, the maximal inclinations of the tracker on the plane X-Y fell in the range of (+15°, −15°) (in most cases, the subjects had only the forward-leaning posture, instead of obvious inclination on left or right). Therefore, θ(t) in Equation (1) can be obtained by integrating the angular velocity (rad/s) of the pitch direction (plane X-Z) from the gyroscope.

The drift of the gyroscope signals should be corrected before data integration. Here, we used a complementary filter [23,24,28] to remove the drift from the gyroscope data. The complementary filter calculates the rotation angle of the sensor by using the accelerometer and gyroscope data. This procedure is based on the fact that the gyroscope responds well to high-frequency-domain signals, and the accelerometer responds well to low-frequency-domain signals [24,27]. Thus, we have:

(2) $$\theta \left(t\right)=\alpha \times \left(\theta \left(t-1\right)+\left(Gyro_{y}\left(t\right)\times dt\right)\right)\;+\;\left(1-\text{ α}\right)\times \theta_{acc}\left(t\right)$$

where α is a given ratio between 0 and 1, $Gyro_{y}\left(t\right)$ is the angular velocity of the gyroscope rotating around the *Y*-axis (i.e., pitch direction), and $\theta_{acc}\left(t\right)$ is the angle calculated from the accelerometer. If the sensor rotates as shown in Figure 5b, then $\theta_{acc}\left(t\right)$ can be obtained as:

(3) $$\theta_{acc}\left(t\right)=\tan^{-1}\frac{a_{z}\left(t\right)}{a_{x}\left(t\right)}$$

where $a_{z}\left(t\right)$ and $a_{x}\left(t\right)$ are the accelerations measured by the sensor along the *Z*-axis and *X*-axis, respectively. In this study, our goal was to detect gait events for calculating temporal parameters. Therefore, Equations (1)–(3) are presented for patterns on one specific axis in stair-walking motions (i.e., the *X*-axis in Figure 2) because the signals of this axis have more significant responses in stair-walking. More detailed sensor fusion and filtering techniques were provided in [28] on three-dimensional orientation estimation for human body parts.

### 2.4. Validation Method and Validation Results

We constructed a two-step staircase in a gait laboratory to validate the proposed method and signals. The participants wore Xsens trackers and walked up and down stairs within the range of a Vicon motion capture system (Vicon, Oxford, OX, UK) [29], which has been described as the gold standard for movement measurement. A reflective marker was attached to the Xsens tracker and the Vicon cameras tracked the marker displacement.

**Figure 6:**
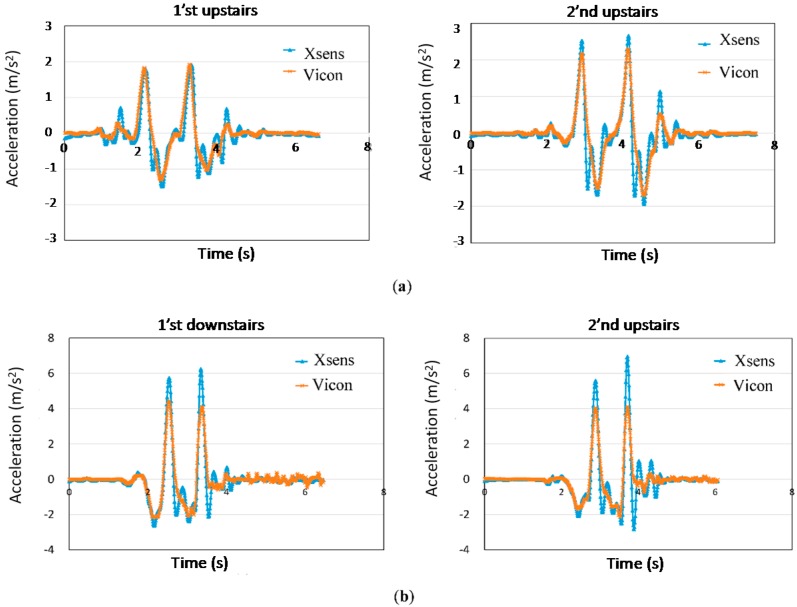
Validation of the Xsens accelerations (**a**) upstairs; and (**b**) downstairs with Vicon data.

We first validated the tracker by asking the child to walk upstairs and then downstairs. The acceleration data from the Xsens tracker were extracted and graphed for comparison with the data collected from the Vicon system. Note that the Vicon system does not measure the acceleration directly. Thus, the displacement of the marker was differentiated twice to produce the acceleration function. Figure 6a shows the upstairs validation, and Figure 6b shows the downstairs validation. Overall, the acceleration signals from the Xsens tracker matched the acceleration functions from the Vicon system. For the upstairs testing, the waveforms from the two systems were almost synchronized. For downstairs walking, although the Xsens signals exhibited higher peak values, both curves were highly correlated. This result shows that we successfully eliminated the influence of gravity from the acceleration (Equation (2)).

Second, we asked the child to walk upstairs and downstairs continually for four rounds. We first detected the peak points of the signals from the Xsens tracker, and compared them with the real heel-contact events detected by the Vicon system. Table 1 shows a comparison of the average time between two sequential peak points and the average time of two sequential heel contacts. The results showed that the error rates were lower than 3%.

**Figure 7:**
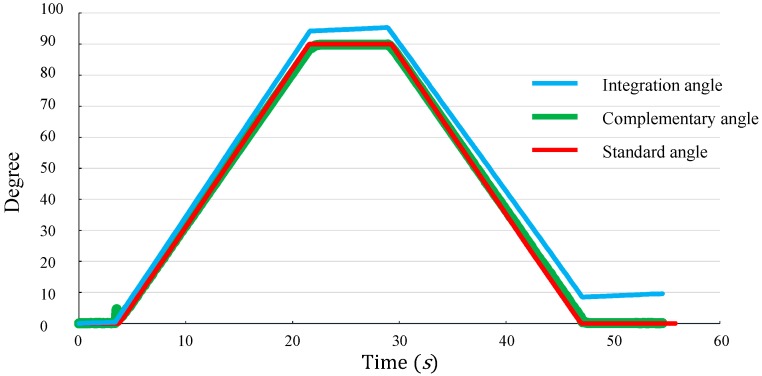
Effect of gyroscope rotation validation. The first curve was calculated by integrating the rotation velocity of the gyroscope, the second curve was calculated by using the complementary filter (Equation (3)), and the third curve was the angle of the step motor (gold standard).

Lastly, we verified that drift was eliminated from the gyroscope. An Xsens tracker was mounted on a step motor. The step motor rotated from 0° to 90° and then rotated back to 0°. Figure 7 shows comparisons of the rotation angles obtained from the gyroscope with the angles obtained from the Vicon system. The first curve in Figure 7 was calculated by integrating the rotation velocity of the gyroscope output, the second curve was calculated by using the complementary filter (Equation (3)), and the third curve was the angle of the step motor (gold standard). As the figure shows, the first curve exhibits a significant drift effect after a certain time. A 10° bias was generated after the motor stopped. Furthermore, the complementary filter of the third curve shows that the drift can be eliminated effectively.

## 3. Results and Discussion

### 3.1. Ten-Meter Straight-Line Walking

**Figure 8:**
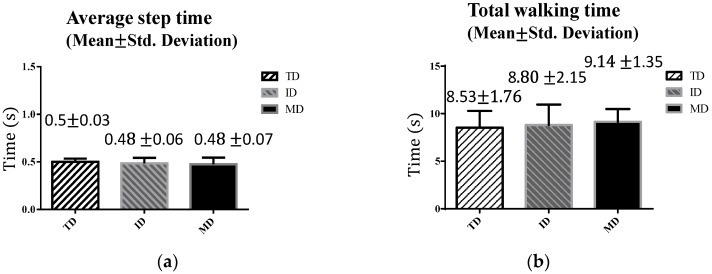
Results of 10-m straight-line walking: (**a**) average step time; and (**b**) total walking time.

In the straight-line walking test, we compared two parameters, namely the average step time (s), and the total walking time (s), among the three groups (TD, ID, and MD) (cf. Figure 3). The results are shown in Figure 8. The TD adolescents generally had larger steps. Thus, their average step time was longer and their total walking time was shorter than those of the other two groups, as shown in Figure 8a,b. The adolescents with MD walked with smaller steps and required the longest total walking time. An ANOVA test was applied to compare the group differences in Figure 8. Table 2 reveals that neither the average step time nor the total walking time reached statistical significance. This indicates that the straight-line walking test cannot effectively distinguish the walking ability levels of TD adolescents from those of adolescents with ID and MD.

### 3.2. Stair-Walking

#### 3.2.1. Upstairs

**Figure 9:**
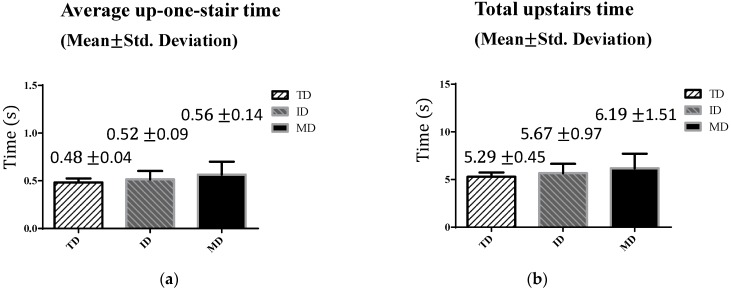
Upstairs walking results: (**a**) average up-one-stair time; and (**b**) total upstairs time.

Figure 9 shows the upstairs walking performance levels of three groups. We compared two parameters: the average up-one-stair time and the total upstairs time, as defined in Figure 4. Unlike straight-line walking, Figure 9 shows that, on average, the adolescents with ID and MD required more time to finish the test than the TD adolescents did. Despite these differences, all adolescents could walk one step in less than 1 s on average. In addition, the standard deviations for the ID and MD groups were higher than that of the TD group, which implied that the two groups demonstrated greater variability during stair-walking.

Table 3 shows the ANOVA results. Scheffé’s method was used for the post hoc test (Table 4). The results reveal that a significant mean difference existed only between the MD and the TD groups (i.e., the rows highlighted). The results suggest that the upstairs walking test is sensitive only for the comparison of participants with MD with TD participants.

#### 3.2.2. Downstairs

**Figure 10:**
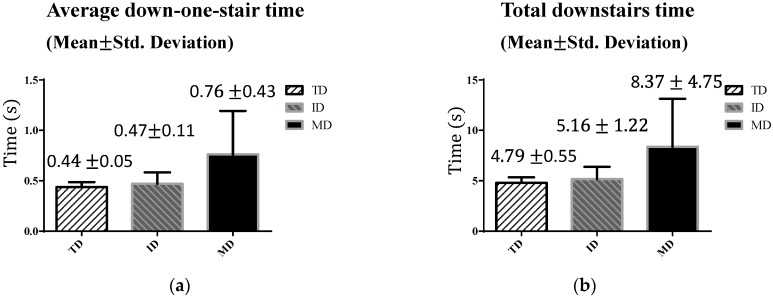
Downstairs walking results: (**a**) average down-one-stair time; and (**b**) total downstairs time.

Figure 10 shows the downstairs walking parameters of the three groups. On average, the TD adolescents and the adolescents with ID required less time to walk down one stair (i.e., <0.5 s) and all stairs (i.e., <6 s). However, the adolescents with MD showed relatively longer down-one-stair time (Figure 10a) and, thus, relatively longer total time downstairs (Figure 10b). The average time required for the adolescents with MD to complete 10-step stair-walking was 1.7 times of the TD adolescents (4.79 s). In addition, the MD group demonstrated greater variability.

Table 5 shows the ANOVA results of the downstairs walking test. The results support the previous finding that significant differences exist among these three groups. Post hoc tests indicated that differences existed between the MD and the TD groups, and between the MD and the ID groups (Table 6). This indicated that downstairs walking is more challenging for adolescents with MD.

#### 3.2.3. Discussion

The results in this study show that the stair-walking test is more sensitive in differentiating adolescents with MD from TD adolescents than the straight-line walking test. During the upstairs and downstairs stair-walking tests, at least one group demonstrated a significant difference of the mean from the other groups. These results were not observed in the straight-line level walking test. Stair-walking, compared with straight-line level walking, imposes greater loads on the musculoskeletal and cardiopulmonary systems; therefore, it was substantially more challenging for adolescents with disabilities.

In addition to the longer durations, these adolescents also demonstrated higher variability during stair-walking. Subjectively, some adolescents demonstrated ancillary movement patterns, including forward leaning, outreached arms, and tap-stepping. The authors of [16] mentioned that those patterns may be a natural means of lowering the center of gravity and increasing the perception of a safety zone for the action. During the walking test, some adolescents tended to raise their hands to touch the handrail or the wall. This response may be attributable to a lack of balance, or a fear of falling. The authors of [14] reported that adolescents with ID often have the “tap-step” pattern, in which the adolescents put two feet on the same stair step when walking. The participants in this study developed different ancillary movement patterns to adapt to the stairs on the basis of their individual statuses.

This study also revealed that downstairs walking was more challenging than upstairs walking for the adolescents with MD. Their functioning seems to be decisively affected not only by the mere existence of a physical and/or cognitive disability but also by its severity. Severe physical disabilities cause physical exhaustion or inadequate physical condition [30]. A significant difference was found between the two groups in ADL (*F* = 1.338, *p* = 0.029). Lack of adaptive ability limits a child’s response strategy for maintaining a stable posture effectively [7]. In addition, Mensch et al. [31] found a positive interaction effect between balance and baseline ADL. During downstairs movement, a subject might need to lower his or her body in a controlled manner to avoid acceleration added by gravity. The results of this study suggest that stair-walking, especially downstairs walking, can differentiate adolescents with MD from TD adolescents and adolescents with ID. This finding can be applied in facilitating clinical differential diagnosis and monitoring training progress in adolescents with MD. This issue can be addressed in future works.

In this study, only one sensor on the lower back was used for measurement. Wherever possible, more sensors should be applied on the limbs or other body segments to capture other movement features. However, compromise is necessary when studying participants with disabilities because more sensors imply longer testing time, which might deteriorate the performance of the children.

So far, the studies of using the inertial sensors to measure the stair-walking performance for the adolescents with ID are limited [25]. The studies of [32,33] addressed the stair-walking performance measurement by using the inertial sensors, but they targeted children with cerebral palsy. The study of [16] proposed a stair-walking intervention strategy, but they targeted children with Down’s syndrome by using a camera. With more and more applications developed by using the inertial sensors, the measurement model used in this paper can also be extended to other subject groups.

## 4. Conclusions

In this study, we applied the motion sensor to the adolescents with intellectual disabilities for measuring their stair-walking performance. The contribution of this work has two aspects. First, we eliminated the gravity effect and the signal drift problem from the sensor to calibrate the results. Second, our work shows that the stair-walking testing can illustrate more significant motion impairment, compared with the conventional straight-line walking test. In the future, we hope to increase the number of subjects to validate these results, and look for the chance of applying the proposed approach to regular interventions.

## Figures and Tables

**Table 1:** Comparison of the peak points with the heel-contact events.

Experiments	Average Time between Two Peaks in 10 m Straight-Line Level Walking			Average Time between Two Peaks in Stair-Walking					
				Upstairs			Downstairs		
	Xsens (s)	Vicon (s)	Error (%)	Xsens (s)	Vicon (s)	Error (%)	Xsens (s)	Vicon (s)	Error (%)
First	1.04	1.05	0.95	1.11	1.11	0.00	1.17	1.16	0.86
Second	0.88	0.87	1.38	1.22	1.19	2.52	0.81	0.83	2.64
Third	0.94	0.96	0.73	1.20	1.23	2.44	0.83	0.84	0.60
Mean	0.95	0.96	1.04	1.18	1.18	0.00	0.94	0.94	0.00

**Table 2:** ANOVA test of 10-m straight-line level walking for three groups.

ANOVA						
		Sum of Squares	*df*	Mean Square	*F*	*p-*Value *
Total Walking Time	Between Groups	3.067	2	1.534	0.453	0.638
	Winthin Groups	199.880	59	3.388		
Step Time	Between Groups	0.006	2	0.003	1.174	0.316
	Winthin Groups	0.146	59	0.002		

* The mean difference is significant at the 0.05 level.

**Table 3:** ANOVA test of upstairs walking for three groups.

ANOVA						
		Sum of Squares	*df*	Mean Square	*F*	*p-*Value *
Total Upstairs Time	Between Groups	6.464	2	3.232	4.256	**0.019**
	Winthin Groups	44.808	59	0.759		
Average Up-one-stair Time	Between Groups	0.053	2	0.026	4.286	**0.018**
	Winthin Groups	0.364	59	0.006		

* The mean difference is significant at the 0.05 level.

**Table 4:** Post hoc tests of upstairs walking for three groups.

Dependent Variable	Disorders Type (I)	Disorders Type (J)	Mean Difference (I-J)	*p*-Values *	95% Confidence Interval	
					Lower Bound	Upper Bound
Total Upstairs Time	TD	ID	−0.37869	0.320	−1.0025	0.2451
	TD	MD	−0.89419	**0.023**	−1.6870	−0.1013
	ID	MD	−0.51550	0.319	−1.3631	0.3321
Average Up-one-stair Time	TD	ID	−0.03319	0.340	−0.0894	0.0230
	TD	MD	−0.08119	**0.022**	−0.1526	−0.0098
	ID	MD	−0.04800	0.295	−0.1243	0.0283

* The mean difference is significant at the 0.05 level.

**Table 5:** ANOVA test of downstairs walking for the three groups.

ANOVA						
		Sum of Squares	*df*	Mean Square	*F*	*p-*Value *
Total Downstairs Time	Between Groups	100.825	2	50.412	12.334	**0.000**
	Winthin Groups	241.147	59	4.087		
Average Down-one-stair Time	Between Groups	0.831	2	0.415	12.320	**0.000**
	Winthin Groups	1.989	59	0.034		

* The mean difference is significant at the 0.05 level.

**Table 6:** Post hoc tests of downstairs walking for the three groups.

Dependent Variable	Disorders Type (I)	Disorders Type (J)	Mean Difference (I-J)	*p*-Values *	95% Confidence Interval	
					Lower Bound	Upper Bound
Total Downstairs Time	TD	ID	−0.37181	0.813	−1.8190	1.0753
	TD	MD	−3.58081	**0.000**	−5.4201	−1.7415
	ID	MD	−3.20900	**0.001**	−5.1753	−1.2427
Average Down-one-stair Time	TD	ID	−0.03412	0.809	−0.1656	0.0973
	TD	MD	−0.32512	**0.000**	−0.4922	−0.1581
	ID	MD	−0.29100	**0.001**	−0.4696	−0.1124

* The mean difference is significant at the 0.05 level.

## Abbreviations

The following abbreviations are used in this manuscript:

TD	Typically Developed
ID	Intellectual Disabilities
MD	Multiple Disabilities
